# The burden and treatment of HIV in tuberculosis patients in Papua Province, Indonesia: a prospective observational study

**DOI:** 10.1186/1471-2334-10-362

**Published:** 2010-12-24

**Authors:** Gysje J Pontororing, Enny Kenangalem, Dina B Lolong, Govert Waramori, Sandjaja, Emiliana Tjitra, Ric N Price, Paul M Kelly, Nicholas M Anstey, Anna P Ralph

**Affiliations:** 1 Menzies School of Health Research-National Institute of Health Research and Development Research Program, Timika, Papua, Indonesia; 2 National Institute of Health Research and Development, Jakarta, Indonesia; 3 Public Health & Malaria Control Department, PT Freeport Indonesia, Timika, Papua, Indonesia; 4 Global Health Division, Menzies School of Health Research, Darwin, Australia; 5 National Centre for Epidemiology and Population Health Research, Australian National University, Canberra, Australia; 6 District Health Authority, Timika, Papua, Indonesia; 7 Department of Infectious Diseases, Royal Darwin Hospital, Darwin, Australia; 8 Centre for Tropical Medicine, Nuffield Department of Clinical Medicine, University of Oxford, UK

## Abstract

**Background:**

New diagnoses of tuberculosis (TB) present important opportunities to detect and treat HIV. Rates of HIV and TB in Indonesia's easternmost Papua Province exceed national figures, but data on co-infection rates and outcomes are lacking. We aimed to measure TB-HIV co-infection rates, examine longitudinal trends, compare management with World Health Organisation (WHO) recommendations, and document progress and outcome.

**Methods:**

Adults with newly-diagnosed smear-positive pulmonary TB managed at the Timika TB clinic, Papua Province, were offered voluntary counselling and testing for HIV in accordance with Indonesian National Guidelines, using a point-of-care antibody test. Positive tests were confirmed with 2 further rapid tests. Study participants were assessed using clinical, bacteriological, functional and radiological measures and followed up for 6 months.

**Results:**

Of 162 participants, HIV status was determined in 138 (85.2%), of whom 18 (13.0%) were HIV+. Indigenous Papuans were significantly more likely to be HIV+ than Non-Papuans (Odds Ratio [OR] 4.42, 95% confidence interval [CI] 1.38-14.23). HIV prevalence among people with TB was significantly higher than during a 2003-4 survey at the same TB clinic, and substantially higher than the Indonesian national estimate of 3%. Compared with HIV- study participants, those with TB-HIV co-infection had significantly lower exercise tolerance (median difference in 6-minute walk test: 25 m, p = 0.04), haemoglobin (mean difference: 1.3 g/dL, p = 0.002), and likelihood of cavitary disease (OR 0.35, 95% CI 0.12-1.01), and increased occurrence of pleural effusion (OR 3.60, 95% CI 1.70-7.58), higher rates of hospitalisation or death (OR 11.80, 95% CI 1.82-76.43), but no difference in the likelihood of successful 6-month treatment outcome. Adherence to WHO guidelines was limited by the absence of integration of TB and HIV services, specifically, with no on-site ART prescriber available. Only six people had CD4+ T-cell counts recorded, 11 were prescribed co-trimoxazole and 4 received ART before, during or after TB treatment, despite ART being indicated in 14 according to 2006 WHO guidelines.

**Conclusions:**

TB-HIV co-infection in southern Papua, Indonesia, is a serious emerging problem especially among the Indigenous population, and has risen rapidly in the last 5 years. Major efforts are required to incorporate new WHO recommendations on TB-HIV management into national guidelines, and support their implementation in community settings.

## Background

Tuberculosis (TB) and HIV infections are major global health threats. An estimated 1.4 million new TB cases in HIV-positive individuals were reported in 2007 [1]. Wide-ranging diagnostic, management and economic challenges are posed by TB-HIV co-infection. HIV confers the greatest risk for TB, increasing the risk of latent TB reactivation 20-fold [2,3]. TB is a leading cause of death among people with HIV [4], and TB patients co-infected with HIV (HIV+/TB+), especially in the absence of antiretroviral therapy (ART), have significantly worse outcomes than those without HIV (HIV-/TB+) [5,6]. TB treatment regimens appropriate for HIV-negative people, such as thrice-weekly therapy during the intensive phase, and a 6-month rifampicin duration, may be suboptimal in HIV-positive people [7].

Priorities in addressing TB-HIV co-infection include instituting the World Health Organisation (WHO)'s '3Is' (Intensified TB case-finding, Isoniazid preventive therapy and Infection control) [8], ensuring routine ascertainment of HIV status in people with TB [9], close integration of TB and HIV services [9,10], and universal access to ART in HIV-positive people [11], including early ART initiation in TB-HIV co-infection [10,12-14]. Acknowledging the improved outcomes achieved with ART [14], WHO guidelines have moved beyond 2006 recommendations guided by CD4 count [10] to now recommend ART in all people with TB-HIV co-infection [13].

Despite these rapid advances in knowledge, substantial barriers persist to the achievement of optimised TB-HIV management goals, especially in lower-income countries. In tackling the challenge of HIV in people with TB, multifactorial barriers include failures to implement HIV testing, failure to prescribe ART or other elements of HIV care, pre-treatment loss to care and post-ART-initiation loss to follow up [15].

In Indonesia, HIV infection rates in TB are not routinely reported [16]. A 2006 study utilising unlinked, anonymous testing in people with TB in Yogyakarta found an HIV seroprevalence rate among TB patients of 1.9% [17]. The overall national estimate is 3% [1]. Papua Province has long been recognised as having one of the highest HIV burdens in Indonesia [18]. An 'Integrated Bio-Behavioral Surveillance' study in 2006, of 6305 Papua Province residents aged 15-49 years, reported population HIV seroprevalence as 2.4% [19]. This survey also revealed low levels of health knowledge: 51.8% overall had heard of HIV/AIDS (26.3% among those with no or limited education). Only 35.4% knew that condom use was protective [19]. Especially high HIV rates of 26% have been documented in Papuan female commercial sex workers [18].

Indonesia has had a policy to provide free ART since 2003 [20]. However, national capacity to widely roll out ART and provide other elements of HIV care is limited [18,20,21]. HIV voluntary counselling and testing was taken up by just 4% of TB patients in one 2006 Indonesian study [17]. Recent estimates of ART accessibility indicate that only about 24% of Indonesian people with advanced HIV infection receive ART; this figure is as low as 3% in Papua Province [20,21].

Our aims were to describe current TB-HIV epidemiology and management in Timika, Papua Province, Indonesia, in order to tailor future interventions. Specifically, we aimed to investigate the burden of HIV infection among adults with smear-positive pulmonary TB, to examine changes over time, to describe current HIV management and compare with 2006 WHO guidelines [9], and investigate TB treatment outcomes among TB-HIV co-infected people.

## Methods

### Study setting

The study was conducted at a community TB clinic in Timika, Papua Province, Indonesia. Timika is adjacent to a major gold and copper mine, and has one of the highest rates of growth of any urban community in Indonesia, from an estimated population of 3000 in 1967 to 200 000 in 2009 [22]. Estimated TB incidence is 311/100 000 [23], compared with 228/100 000 for Indonesia overall [1]. The Timika population currently comprises approximately half Indigenous Papuans and half non-Papuan Indonesians.

### Participants

Adult outpatients (>15 years) with newly-diagnosed sputum smear positive pulmonary TB who were not pregnant, agreed to stay in Timika for the study duration, and gave written informed consent, were eligible for enrolment. Study participants were recruited June 2008-October 2009 as part of an ongoing trial of adjunctive therapies in TB (see http://clinicaltrials.gov/show/NCT00677339), and followed weekly for 8 weeks, then monthly for 24 weeks. Information available after 24 weeks regarding HIV care was also recorded. Ethnicity was documented as Indigenous Papuan or Non-Papuan. Longitudinal trends were determined by comparing current with previously-collected data from Timika (an observational study of 112 smear-positive pulmonary TB patients in 2003-4), results of which are published elsewhere [23-26].

### HIV testing

Voluntary counselling and testing (VCT), including detailed education about HIV/AIDS, was conducted in accordance with Indonesian national guidelines by a doctor at the TB clinic, or a trained counsellor at the adjacent sexual health clinic. Consultations were either in private, or with a spouse/family member/guardian if requested by the patient. Study participants could decline testing, or demonstrate agreement by signing or applying their thumbprint to the VCT consent form. The process took 30-60 minutes.

HIV antibody was tested using SD BioLine HIV-1/2 3.0™ antibody test (Standard Diagnostics, Inc). If positive, two confirmatory rapid tests were performed (Abbott Determine™ HIV-1/2 [Inverness Medical], and Oncoprobe™ [PT Oncoprobe Utama]). These tests have high reported sensitivity and specificity [27,28]. A fluorescence-activated cell sorter was used for CD4+ T-cell assays, when available.

### Clinical and laboratory assessments

Baseline and follow-up evaluations included: documentation of clinical history and symptoms, body mass index (BMI), forced expiratory volume in one second (FEV_1_, performed using MicroLoop spirometer, MicroMedical), 6-minute walk test (6 MWT: distance walked in 6 minutes on a straight walking track) measured according to American Thoracic Society guidelines [29], St George's Respiratory Questionnaire (SGRQ: a health-related quality of life score) modified slightly to reflect local conditions and translated into Indonesian language [26,30], chest radiography (standard full-size posteroanterior chest x-ray), and haemoglobin measured using point-of-care iSTAT^® ^tests. Anaemia was defined as haemoglobin <13.5 g/dL in males and <11.5 g/dL in females. Documented chest x-ray findings included percentage of abnormal visible lung fields, presence of cavitation, effusion or miliary disease, and an overall score comprising percentage of lung affected plus a weighting of 40 if cavitation was present, as described elsewhere [31]. Sputum microscopy was performed at the onsite laboratory, with density of acid fast bacilli (AFB) graded as 1, 2 or 3+ according to standard protocols [32]. Smear negativity was defined as the time point (week after treatment) at which the first of 3 consecutive negative results was recorded, or the first negative result if <2 subsequent results were recorded [33,34]. Pulmonary function was defined as moderately impaired if FEV_1 _60-69% predicted, and moderately to severely impaired if 50-59% predicted [35]. Unfavourable outcomes were defined as death, life-threatening illness or hospitalisation.

### Reference guidelines

WHO 2006 guidelines were used as the best-practice reference, recommending ART initiation within 2-8 weeks after TB treatment commencement for CD4 <200 or unknown, after 8 weeks if CD4 200-350, and deferral of ART if CD4 > 350 [9,10].

### Statistical analyses

Statistical calculations were performed using Intercooled Stata 10.1. Graphs were created in GraphPad Prism 5. Statistical tests were two-sided, with a p-value of <0.05 indicating statistical significance. Intergroup differences in means or medians were compared using 2-sample t-tests or Wilcoxon rank sum tests as appropriate. Differences in proportions were calculated using Pearson's χ^2 ^test or Fisher's exact test as appropriate. Multiple linear regression models were used to determine relationships after controlling for potential confounding factors. Final model goodness-of-fit was assessed using the Hosmer-Lemeshow test. Kaplan-Meier survival analysis was used to examine time-to-event data (time to sputum smear conversion using weekly sputum smear readings). Patient subgroup analysis was performed by Cox regression (proportional-hazards) models and hazard ratios and 95% confidence intervals.

### Ethics

Approval was granted by the ethics committees of the National Institute of Health Research and Development (Jakarta, Indonesia), Menzies School of Health Research (Darwin, Australia) and the Australian National University (Canberra, Australia). Written informed consent was obtained from participants in Bahasa Indonesia, and oral translation was provided as required in an appropriate Papuan language.

## Results

One hundred and sixty two smear-positive pulmonary TB patients were recruited. One hundred and forty one (87.0%) were offered VCT. Uptake of HIV testing was high (138/141 = 97.9%); only 3 participants, all female, declined an HIV test. Eighteen of 138 people (13.0%) who had an HIV antibody test were HIV positive, confirmed with 3 rapid assays. See Table 1.

**Table 1:** Characteristics of 138 study participants with known HIV status

	All	HIV+	HIV-	p value (HIV+ vs HIV-)
Number (%)	138	18 (13.0)	120 (87.0)	

Age in years: median (range)	27 (15-65)	31 (16-60)	27 (15-56)	1.0

Papuan: no. (%)	67 (48.6)	14 (77.8)	53 (44.2)	**0.01**

Female: no. (%)	43 (31.2)	7 (38.9)	36 (30.0)	0.4

Current smoker: no.(%)	42 (30.4)	5 (27.8)	37 (30.8)	0.8

Highest educational attainment: no. (%)				
No schooling	10 (7.2)	4 (22.2)	6 (5.0)	
Primary school	28 (20.3)	4 (22.2)	24 (20)	0.05*
High school	96 (69.6)	10 (55.6)	86 (71.7)	
Academy or university	4 (2.9)	0 (0)	4 (3.3)	

Unemployed: no. (%)	59 (44.7)	12 (66.7)	47 (41.2)	0.04*

Owns telephone: no. (%)	77 (55.8)	8 (44.4)	69 (57.7)	0.3

**Clinical and laboratory investigations**				

BMI in kg/m^2^: mean (range)	19.3 (12.9-32.5)	19.2 (12.9-26.7)	19.4 (13.3-32.5)	0.8

Percentage of predicted FEV_1_: mean (range)	63.9 (16.6-108.5)	59.5 (16.5-92.0)	64.6 (23.9-108.5)	0.3

6 minute walk test in m: median (range)	415 (0-612)	390 (0-485)	415 (75-612)	**0.04**

St George's Respiratory Questionnaire† total score: median (range)	38.3 (5.2 - 91.9)	41.7 (13.6-67.0)	38.1 (5.2-91.9)	0.4

Haemoglobin in g/dL: mean (range)	12.4 (7.1-16.0)	11.2 (8.5-12.9)	12.6 (7.1-16)	**0.002**
Anaemia: n (%)	77 (55.8)	14 (77.8)	63 (52.5)	**0.053**

White cell count × 10^9^/L: mean (range)	8.0 (1.6-22.7)	6.8 (2.4-13.2)	9.0 (1.6-22.7)	**0.01**

Sputum smear ≥2+: no. (%)	61 (45.2)	5 (27.8)	56 (47.9)	0.1

Chest X-ray				
Total score: median (IQR)	67 (4-140)	57 (19-121)	68 (6-140)	0.5

Cavitary disease on CXR: no. (%)	74 (55.2)	6 (33.3)	68 (58.6)	**0.04**

Pleural effusion: no. (%)	23 (17.2)	7 (38.9)	16 (13.8)	**0.009**

*Differences in education and employment were not significant in multivariate models controlling for ethnicity. †St George's Respiratory Questionnaire results were only available in 119 participants (15 HIV+ and 104 HIV-)

**Figure 1:**
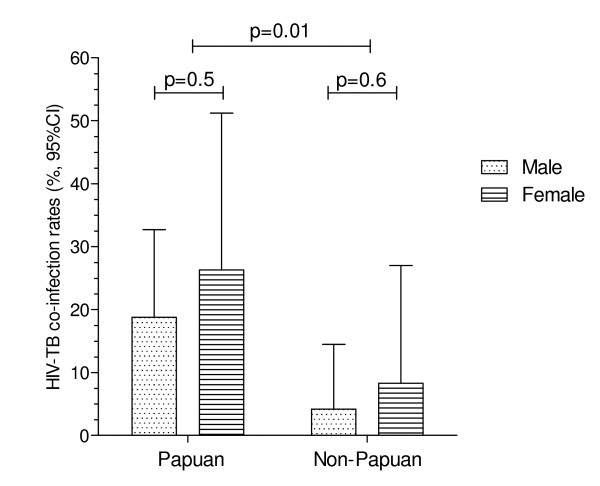
**Rates of TB-HIV co-infection among study participants**. p values calculated using Fisher's exact tests

In total, 21% (14/67) of Papuans were HIV positive compared to 5.6% (4/71) Non-Papuans (OR 4.4 [95% CI: 1.4-14.2], p = 0.02). Papuan women comprised the highest-risk subgroup, although confidence intervals were wide (Figure 1). HIV+/TB+ participants were less likely than HIV-/TB+ to be employed (6/18 = 33.3% vs 67/114 = 58.8%, p = 0.04), and less likely to have achieved an educational level above primary school (10/18 = 55.6% vs 89/119 = 74.8%, p = 0.05). However, these associations were no longer significant after controlling for ethnicity. HIV status was unrelated to age.

### Longitudinal trend in TB-HIV co-infection rate

**Figure 2:**
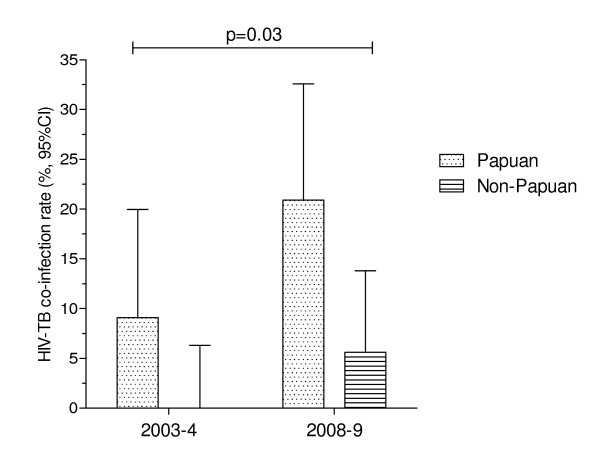
**TB-HIV co-infection rates in Timika in 2003-4 compared with 2008-9**. p value calculated using Fisher's exact test

We conducted a study of smear-positive pulmonary TB patients at the same Timika TB clinic in 2003-2004, in which HIV status was ascertained in all 112 study participants. HIV seroprevalence was 4.5% overall (5/112) [26]. Comparing this 2003-2004 data with the current findings, a significant rise in the rate of TB-HIV co-infection is evident in Timika in the last 4 years (OR = 3.2 [95% CI: 1.2-8.9], p = 0.03, Figure 2).

### Clinical characteristics

Clinical characteristics according to HIV status are presented in Table 1. Study participants with HIV had significantly lower exercise tolerance, walking a median 25 m less than those without HIV (p = 0.04). The mean haemoglobin and white cell counts were significantly lower in HIV+ patients (mean difference = 1.3 g/dl [95% CI 0.5-2.2] p = 0.002, and 2.1 × 10^9^/L [95% CI 0.5-3.8] p = 0.01 respectively), with all HIV+ males, and 3/7 HIV+ females, being anaemic (overall OR 3.2, 95% CI 0.99 - 10.2; Table 1). Pulmonary function was moderately impaired in HIV-/TB+ participants (mean FEV_1 _64.6%), but moderate-severely impaired in the HIV+/TB+ participants (mean FEV_1 _59.5%), however this difference was not statistically significant (p = 0.3, Table 1). Weight or BMI and quality of life scores (SGRQ) did not significantly differ according to HIV status (Table 1). Associations, apart from anaemia, remained the same when controlling for weight, ethnicity and sex.

Chest x-ray findings differed significantly, with cavitary disease being less common (OR = 0.4, 95% CI 0.1-1.0), and pleural effusion (i.e. extra-pulmonary TB) more common (OR = 3.6, 95% CI 1.7-7.6), in the HIV+/TB+ group compared with HIV-/TB+ group. However, overall extent of radiological disease (chest x-ray score) did not differ (Table 1).

### Outcome

HIV status did not significantly influence time to sputum smear conversion (hazards ratio [HR] 0.7, 95% CI 0.41 - 1.3, p = 0.2). The HIV+/TB+ group had a similar overall likelihood of treatment success (HIV+/TB+ cure or completion: 85.7%; HIV-/TB+ cure or completion: 92.6%, p = 0.2). However, they were more likely to suffer an unfavourable outcome, which was reported in 16.7% (3/18) of HIV+/TB+ participants compared with 1.7% (2/120) of the HIV-/TB+ participants (OR 11.8, 95% CI 1.8-76.4). These comprised in the HIV+/TB+ group: 2 deaths (1 progressive respiratory illness due to TB or secondary pneumonia, and 1 stroke complicated by aspiration pneumonia) and 1 hospitalisation (due to pneumothorax and severe malnutrition, BMI 12.9 kg/m^2^), and in the HIV-/TB+ group, no deaths and 2 hospitalisations (1 vomiting/dehydration and 1 multi-drug resistant-TB with progressive pulmonary disease and large pleural effusion). The relationship between HIV status and default rates could not be determined, since people who defaulted often did so prior to HIV testing being offered (of the original 162 study participants, 8 defaulted: of these 3 were HIV negative, none were known to be positive, and 5 had undetermined status).

### HIV Management

CD4 counts were obtained in 6 patients (Table 2). According to 2006 WHO guidelines, 14 people were eligible for early ART initiation (CD4 < 200 cells/μL or unknown), 1 for ART initiation after week 8 (CD4 200-350), and 3 for deferred ART initiation (CD4 > 350). However, during their 6-month TB treatment period, only one person with TB-HIV co-infection was successfully commenced and maintained on ART, 1 patient with known HIV at the time of TB diagnosis was already taking and remained on ART throughout her involvement in the study, and 2 additional patients who had been referred for ART were commenced by the authorised ART prescriber after their TB treatment was completed.

**Table 2:** TB-HIV co-infection management

TB-HIV co-infected study participants	
CD4+ T-cell count: no. (%)	6 (33)
CD4+ T-cells/μL: median (range)	318 (18-739)
CD4+ T-cells <200	2
CD4+ T-cells 200-350	1
CD4+ T-cells >350	3
CD4+ T-cells unknown	12

Anti-retroviral therapy: no. (%)	4/18 (22)
Commenced prior to TB diagnosis	1
Commenced during TB treatment	1
Commenced after TB treatment completed	2

ART type	
AZT/3TC/nevirapine	3
AZT/3TC/efavirenz	1

Co-trimoxazole: no. (%)	11/18 (61)

## Discussion

### TB-HIV co-infection rates

This study has identified that TB-HIV co-infection rates have increased significantly during 5 years in Timika, Indonesia. Among the Indigenous Papuan subgroup, HIV seroprevalence in TB patients rose almost 5-fold to 21%. Although this is still lower than in some global regions (e.g. 51% for the WHO Africa region [1]) it is among the highest reported from Asia, and the rapid rate of change has outstripped local capacity to adequately respond to the problem. HIV co-infection rates differ markedly across Asia, ranging from 0.5% in a large study in Guangxi, China [36], to an estimated 3% overall for Indonesia and 17% in Thailand [1]. Published data from neighbouring Papua New Guinea are scant but similar to rates reported here, with HIV prevalence in incident TB cases estimated at 19% in 2007 [1].

### Differences between HIV positive and negative TB patients

Reasons for ethnic differences in HIV rates were not investigated in this study but may comprise a combination of factors including sexual behaviours, knowledge of sexual health and transmission prevention, and possibly, the difference in male circumcision rates (about 5% of Papuans and 70% of Non-Papuans are circumcised) [19]. Injecting drug use is not thought to contribute to HIV transmission in Papua Province [19]. The trend towards women being at greater risk in Timika is in keeping with findings elsewhere of higher HIV rates in young women compared with same-aged men [37]. HIV+ people were less likely to be educated or employed, indicating that HIV education is required at the community level, not just targeting schools or workplaces.

Clinical differences according to HIV status in this study were not large. A limitation of the study is that the small numbers mean it may be under-powered to adequately detect differences between HIV+ and HIV- groups. No significant differences in symptoms or perceived health-related quality of life were identified. Weight (or BMI) was also not different at baseline. This may indicate that HIV infections overall were not advanced, also supported by the relatively well-preserved CD4+ T-cell counts in some individuals (Table 2). These findings emphasise the importance of routine HIV testing in TB rather than restricting testing to those with additional features suspicious for HIV (such as oesophageal candidiasis), as has been practiced elsewhere [38]. The HIV+/TB+ group did have significantly lower exercise tolerance, and a 1.5-fold increased risk of anaemia. The latter is likely to be multifactorial, with important contributions in the Timika population from impaired nutrition, helminth infection and high rates of multidrug resistant *Plasmodium falciparum *and *vivax *malaria [22]. This illustrates the compounding effect of overlapping endemic illnesses, potentially contributing to other poor health outcomes such as the very high maternal and infant mortality in Timika [39], and known increased mortality risk in patients with TB-HIV co-infection [5].

Lower rates of cavitary disease and higher likelihood of pleural effusion were identified among HIV positive people at TB diagnosis in our study, consistent with previous studies examining chest x-rays in TB-HIV co-infection [40,41]. Since cavitation largely determines sputum bacillary grade [42,43], TB-HIV co-infection is also associated with higher likelihood of smear-negative disease, or low bacillary density in smear-positive disease, in proportion to CD4 count [44]. Our data support these findings, although not reaching statistical significance. Such findings emphasise the difficulty of establishing TB diagnoses (based on sputum smear and radiological appearance) in HIV infection. Problems in confidently excluding active TB in HIV contribute to some persisting reluctance to widely roll-out isoniazid preventive treatment, an important and underutilised strategy for preventing TB in people with HIV/AIDS [8,45]. Indeed, this strategy is not currently routine in Indonesia. Improving diagnostic sensitivity through inexpensive measures in laboratories (e.g. sputum concentration prior to ZN staining, fluorescence microscopy using an inexpensive light-emitting diode light source, or simple culture-based techniques) [12] and clinics (e.g. educating medical staff to appreciate radiological pathology characteristic of TB-HIV co-infection), are not necessarily beyond the means of lower-income settings.

### Barriers to HIV testing and treatment

In this study, HIV status was established in 85% of participants. Although the refusal rate was low, missed opportunities for HIV diagnosis are widely recognised internationally as well as in Timika. For example, the HIV status was reported in only 35% of Australian TB cases in 2006 [46], and nearly half of all TB patients in London were not offered HIV testing in 2003-4 [47]. Barriers to HIV testing in the current study relate primarily to availability of a certified counsellor, availability of a private room, and the time required to provide pre-test counselling. Conducting VCT in a confidential manner is difficult in a crowded TB clinic, reinforcing previous findings of the requirement for the right structural conditions to be present at a clinic to support effective VCT [17]. Given the lack of general knowledge about HIV, reasonably high pre-test probability of a positive result (especially among Papuans), and the degree of stigmatisation suffered by HIV positive individuals [21], the pre-test counselling process in Timika is justifiably lengthy. On identifying HIV positivity, access to appropriate care including ART and condoms is limited; thus the benefit of HIV status knowledge is reduced. Therefore prevailing attitudes among local medical staff has been to take a cautious approach, even if this leads to incomplete ascertainment of HIV status.

Deployment of provider-initiated opt-out HIV testing has been widely advocated [48] as a strategy to improve HIV detection rates [9,10,49-51]. However, a stringent VCT process was introduced in Timika in 2008, stipulating the requirement for written consent, and for only a doctor or professional with specific certification, to conduct VCT. A study investigating barriers to HIV testing among TB patients in another Indonesian province identified both patient and health provider factors as reasons for low VCT uptake [52]. Among patients, these included low HIV knowledge, the disincentive of having to access and endure VCT, and fear of knowing the test results; and among health care providers: low HIV knowledge, communication issues, concern about patients feeling offended, stigmatization and additional work load [52]. Implementing locally-appropriate strategies to improve the proportion of TB patients who are offered HIV testing is an important priority.

Access to CD4+ T-cell testing was limited due to difficulties in maintaining the analyser and lack of staff familiarity with its operation. Only four (5.6%) of the HIV positive study participants successfully commenced ART, 2 after TB treatment completion, even though 15 were eligible for ART during TB treatment according to 2006 guidelines. WHO guidelines on the timing of ART initiation in TB have evolved rapidly on the basis of new trials [14], such that universal ART initiation regardless of CD4 count is now advocated in TB-HIV co-infection [13]. Integration of TB-HIV is strongly advocated [10,13]. However, a chief barrier to ART initiation in Timika is the absence of such integration, with no authorised ART-prescriber available in the vicinity of the TB clinic. In Indonesia, ART can be prescribed only by a designated person at nominated sites (usually hospitals). Even if a patient is successfully referred to, and attends, a designated ART-prescription site, reasons for low ART prescription rates cited by medical staff include concern about potential poor adherence, and drug toxicities or paradoxical reactions. Use of co-trimoxazole preventive therapy was somewhat better, in 11 of 18 patients, as this was able to be prescribed by any doctor. However there is still evidently scope for improved utilisation of this important preventive therapy. Education of healthcare providers about ways to support adherence and manage TB-HIV co-infection is therefore greatly needed.

## Conclusion

Rising HIV rates have become a serious concern in southern Papua. Timika has experienced large recent population fluxes related to the local mining industry attracting migrants from rural areas and other parts of Indonesia, and thus presents a classic demographic scenario for burgeoning HIV rates. The association between HIV and migrant labourers, especially mine workers and a thriving commercial sex industry, has been well established elsewhere [53,54].

These data provide an important South-East Asian perspective on the overlapping TB and HIV epidemics, and illustrate the added morbidity suffered by HIV+ compared with HIV- individuals with TB. This study demonstrates that, despite knowledge of TB-HIV management guidelines and even within the setting of a well-regarded TB Directly Observed Treatment facility, management of TB-HIV co-infection poses an enormous challenge in resource-limited environments. Multiple interventions are now required at community, patient and healthcare provider levels, to address rising HIV rates and the barriers to testing and treatment. The recent publication of revised WHO guidelines on TB-HIV management provides a timely opportunity to incorporate these into national guidelines, and support their implementation in community settings.

## Competing interests

The authors declare that they have no competing interests.

## Authors' contributions

GJP, GW and EK recruited participants, conducted VCT and provided patient care. DBL, ET, S and DAL participated in designing and facilitating the study and provided operational support. APR, PMK and NMA designed and supervised the study and participated in data collection. RNP undertook data management and logistic support. APR performed the data analysis and wrote the first manuscript draft. All authors read and approved the final manuscript.

## Pre-publication history

The pre-publication history for this paper can be accessed here:

http://www.biomedcentral.com/1471-2334/10/362/prepub
